# Supplementary material for: 6mA DNA Methylation on Genes in Plants Is Associated with Gene Complexity, Expression and Duplication
Source: Plants (Basel). 2023 May 10;12(10):1949. doi: 10.3390/plants12101949 (PMC10221889; doi:10.3390/plants12101949)

Supplementary Figure S1. Length distribution and the read quality of SMRT sequencing reads in Indian lotus, Australian lotus, Russian lotus, and Thai lotus genomes.

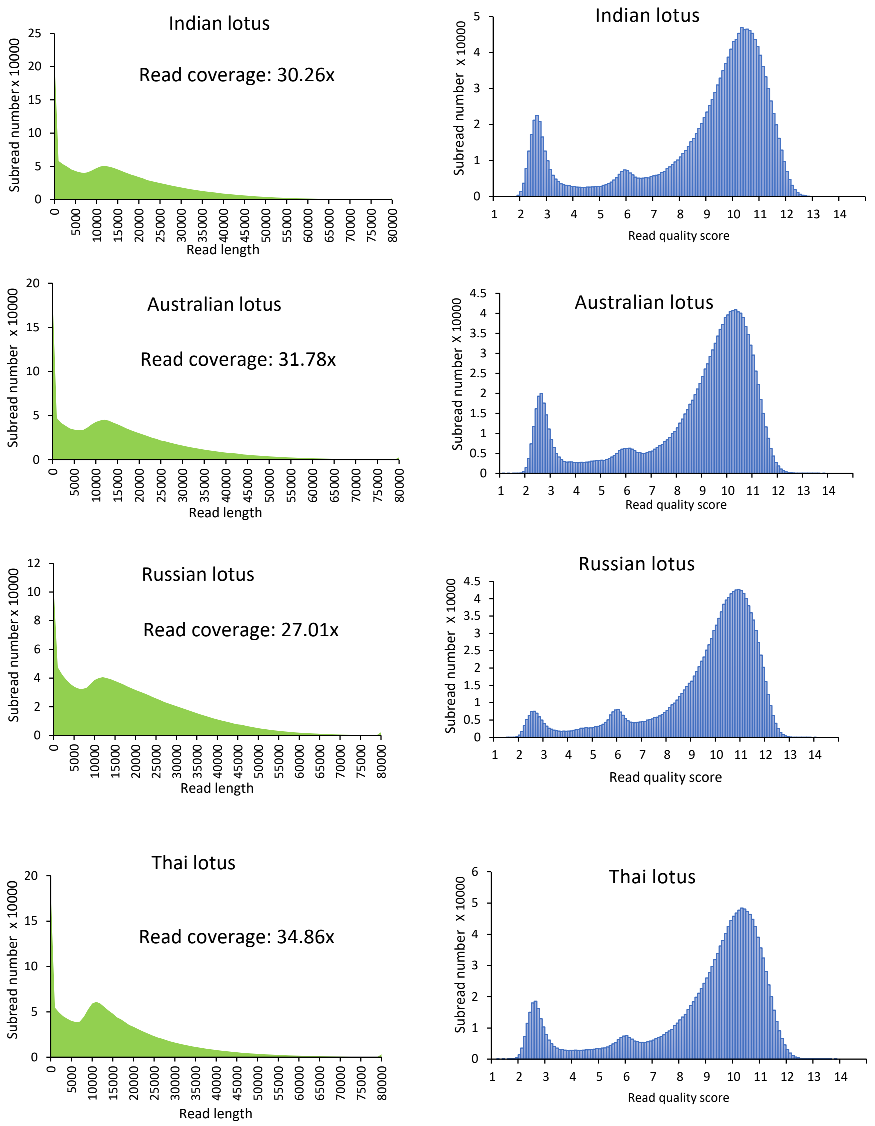

Supplementary Figure S2. Genome assembly and annotation of four wild N. nucifera.A-D. Circos plots of the Indian lotus (A), Australian lotus (D), Russian lotus (C), Thai lotus (D) genome assemblies. Four rings from outside to inside show the chromosomal positions (1st), gene density (2nd), repeat density (3rd), and GC content (4th).

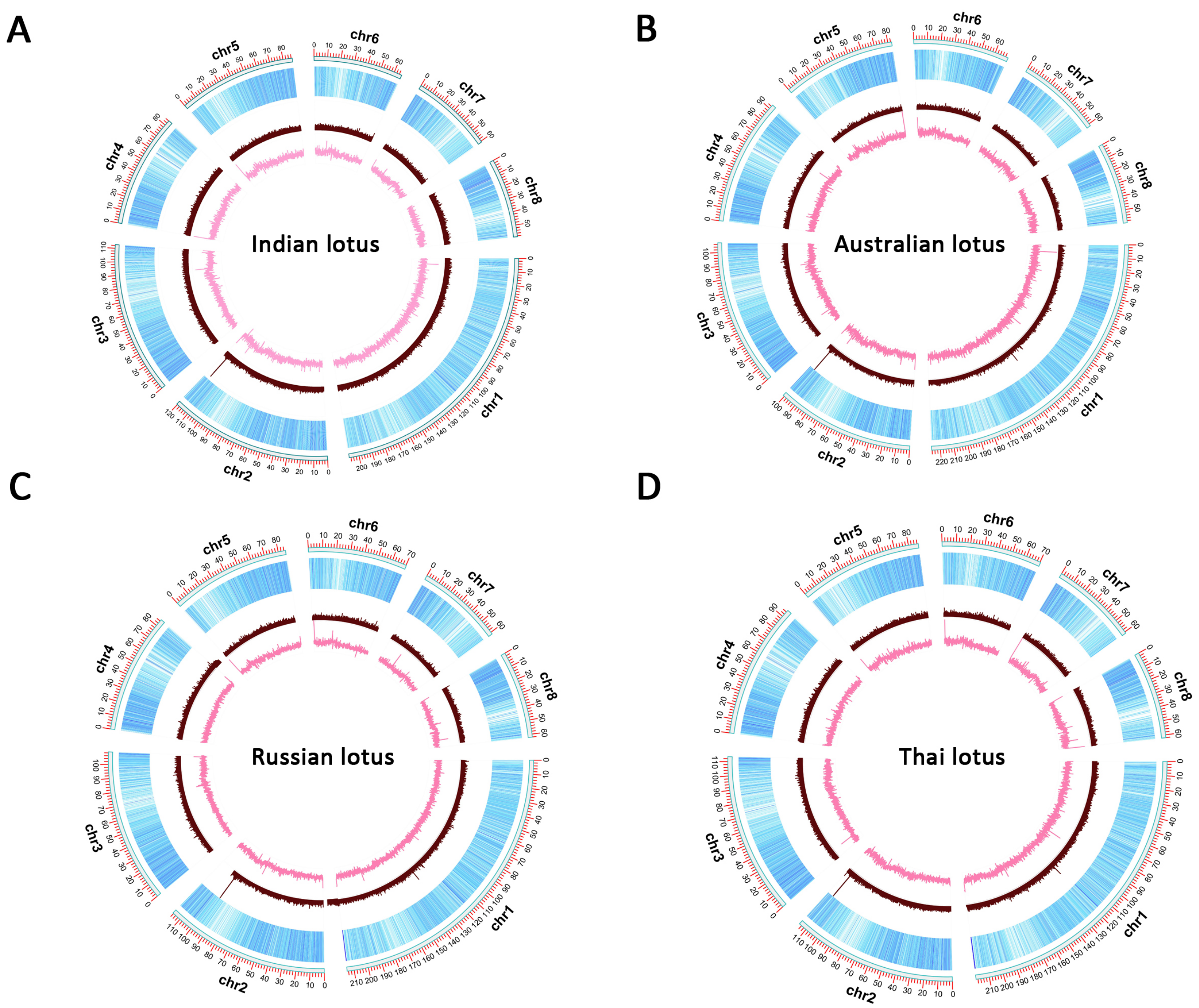

Supplementary Figure S3. Proportion of major classes of repeat families in four lotus genomes. SINEs: short interspersed nuclear elements; LINEs: long interspersed nuclear elements; LTRs: long terminal repeat retrotransposons.

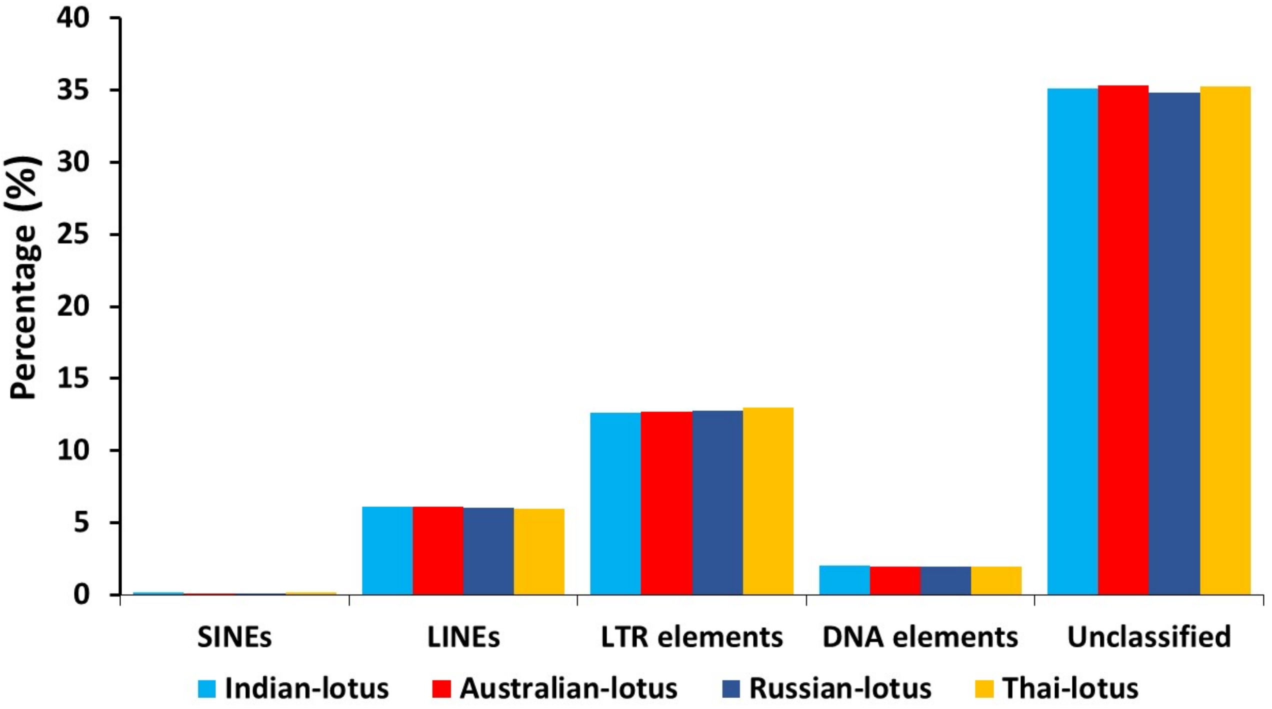

Supplementary Figure S4. The top consensus motif containing 6mA sites ±4bp sequences identified by MEME-ChIP in the four *N. nucifera*. The p-value generated by MEME-CHIP is shown under the logo.

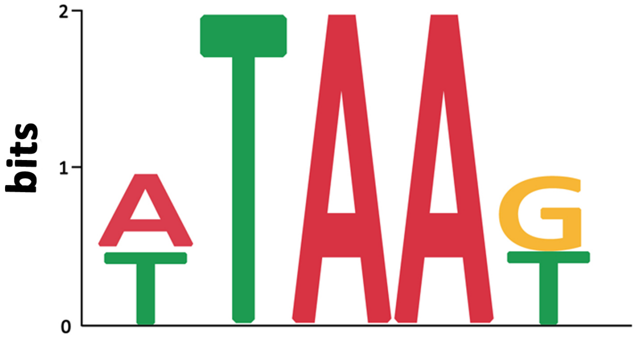

Indian P=6.3e-16265   Russian P=1.4e-19384

Australian P=4.7e-10696   Thai P=5.7e-10887

Supplementary Figure S5. Genome-wide identification of 6mA level in lotus genomes. (A-D). Circos plots of Nanopore-based 6mA and 5mC distribution in Indian lotus (A), Australian lotus (B), Russian lotus (C, and Thai lotus (D) genomes. Four rings from outside to inside show the chromosomal positions (1st), 6mA sites density (2nd), 5mC sites density (3rd), and density of genes with 6mA (4th).

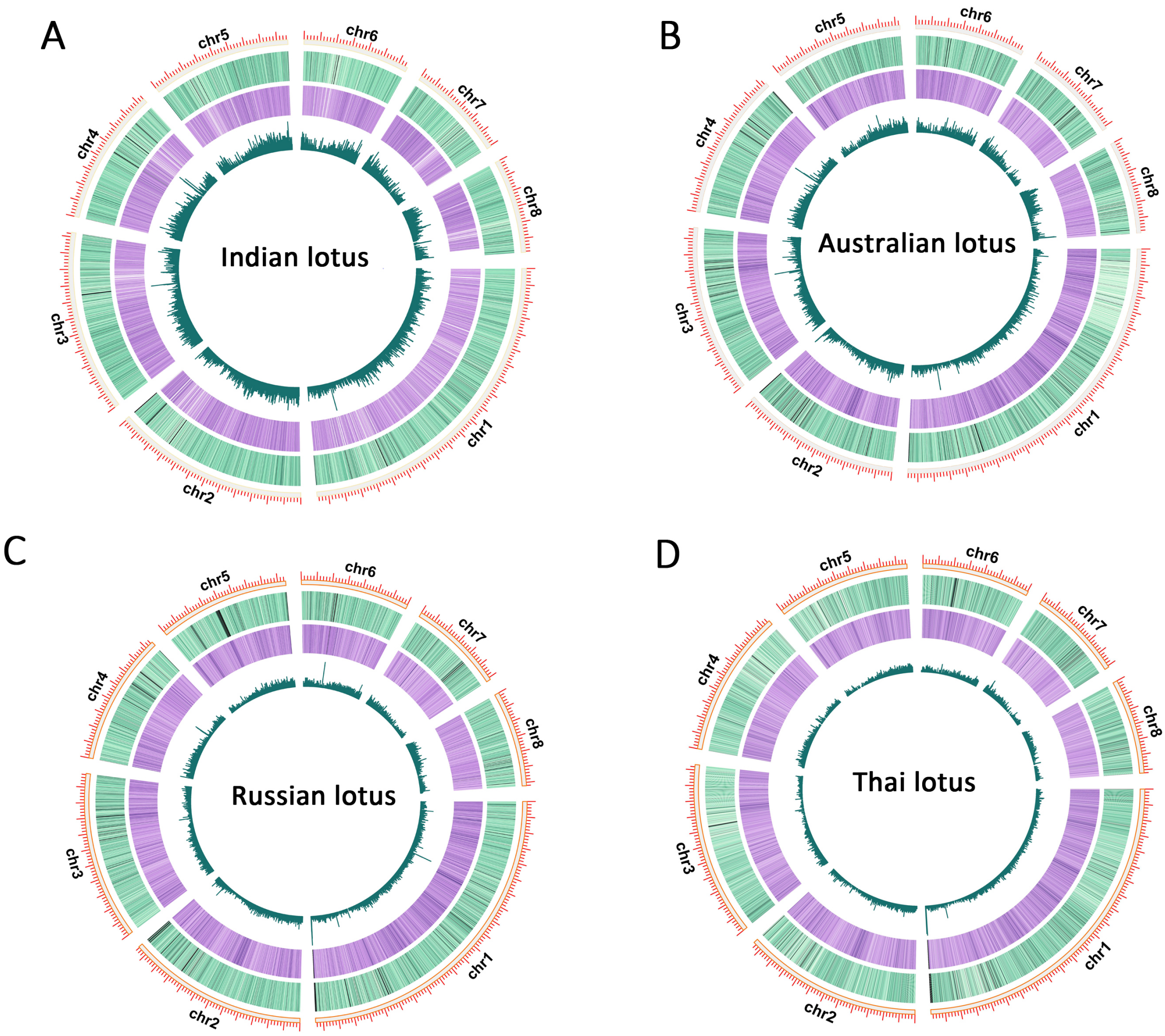

Supplementary Figure S5. 6mA methylation level in three 5mC contexts. (A-C). Percentage of 6mA sites in three differnt 5mC contexts CGN, CHG, and CHH (where N = A, T, C, or G; H = A, T, or C) in Indian lotus (A), Australian lotus (B), Russian lotus (C), and Thai lotus (D) genomes. The different 6mA methylation level between 5mC contexts was tested by chi-squared test, and ** means p-value < 0.01.

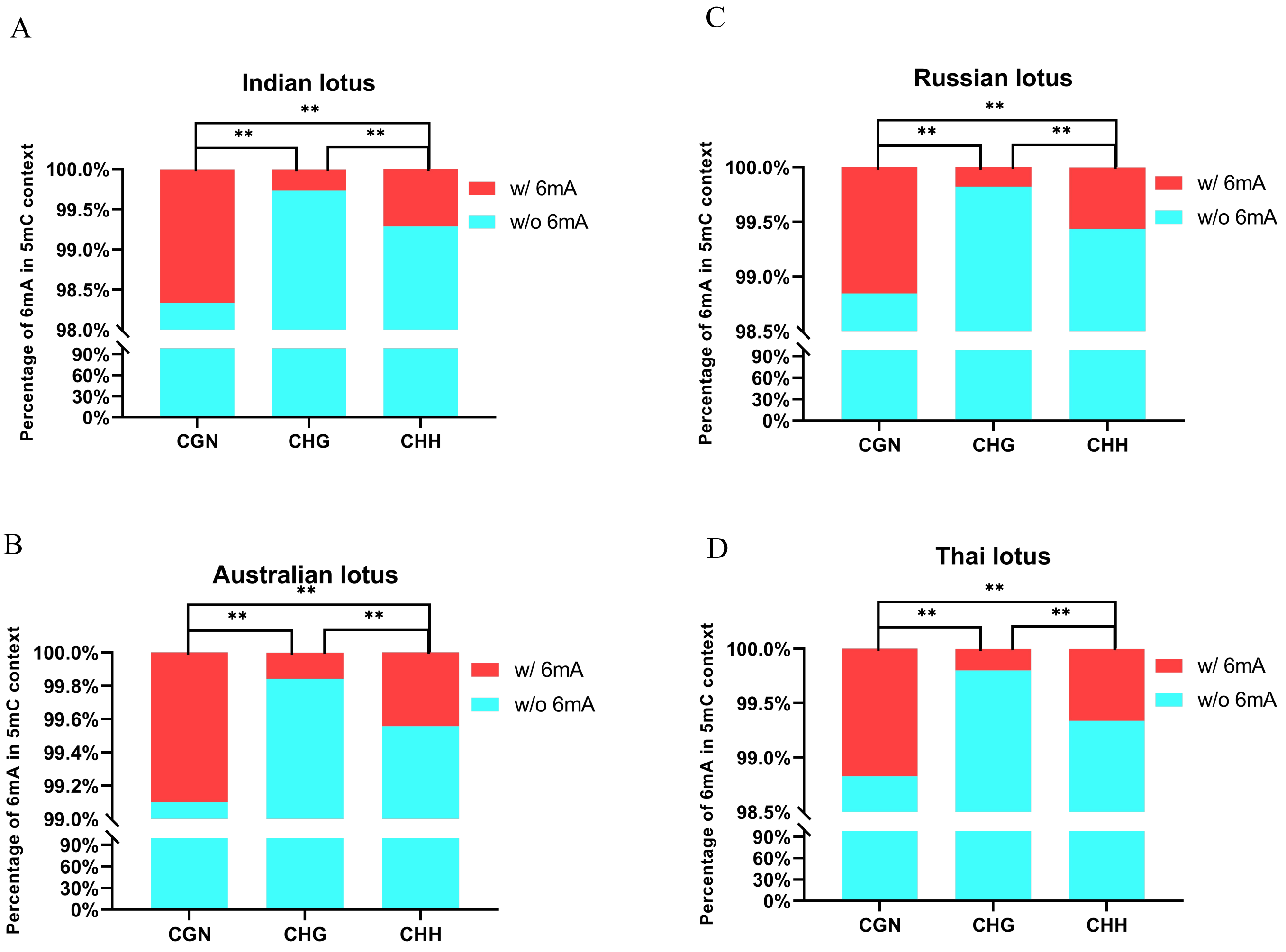

Supplementary Figure S7. The 6mA methylation levels in protein coding genes and transposable elements in four lotus. Bar charts showing the ratio of 6mA sites in the length of protein-coding genes and in the length of transposable elements in the four genomes.

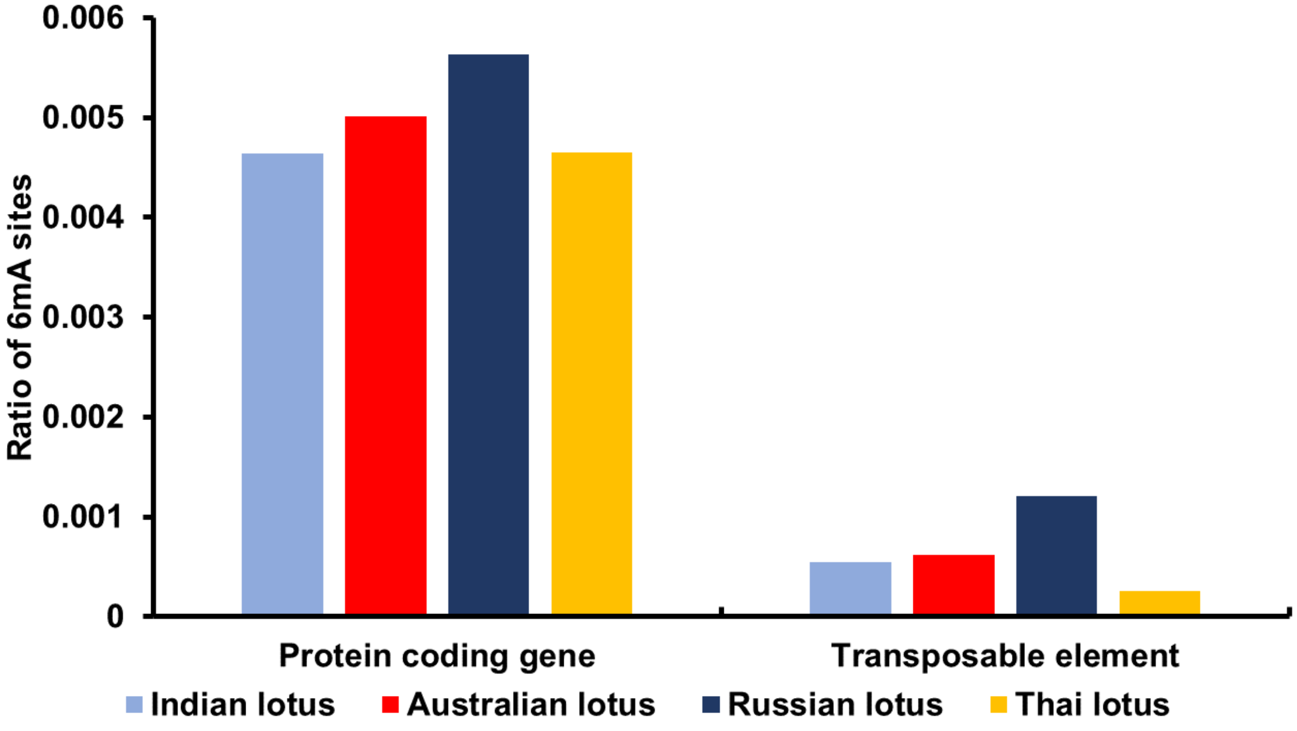

Supplementary Figure S8. Bar graph showing the number of 6mA sites in gene-bodies in Indian lotus, Australian lotus, Russian lotus, and Thai lotus.

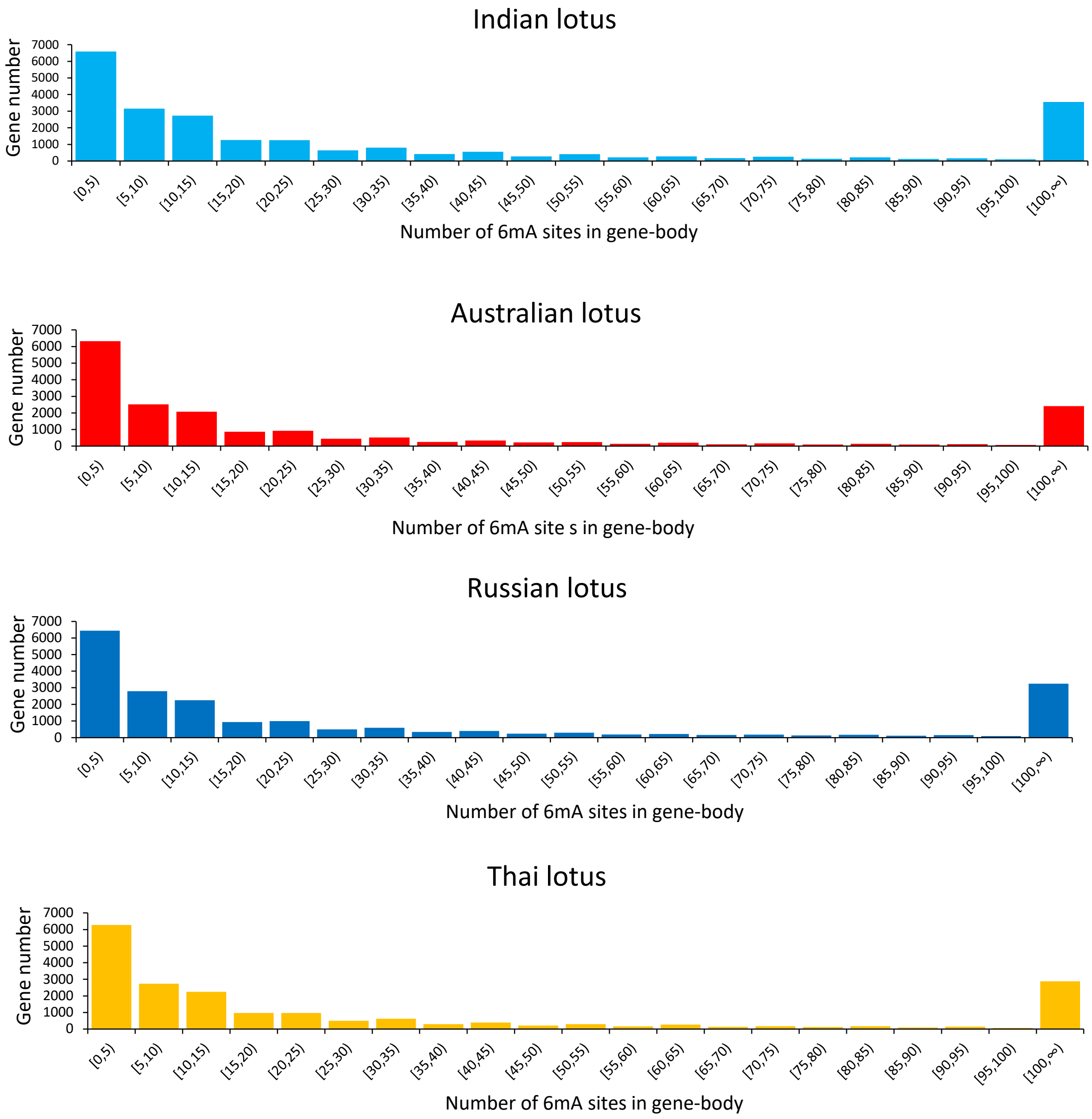

Supplementary Figure S9. Distribution of 6mA sites around the gene start/end site (GSS/GES) in four *N. nucifera* genomes. 6mA occupancy represents the 6mA sites against the total adenine sites in each 50-bp window plotted within 2-kb upstream and downstream of the GES.

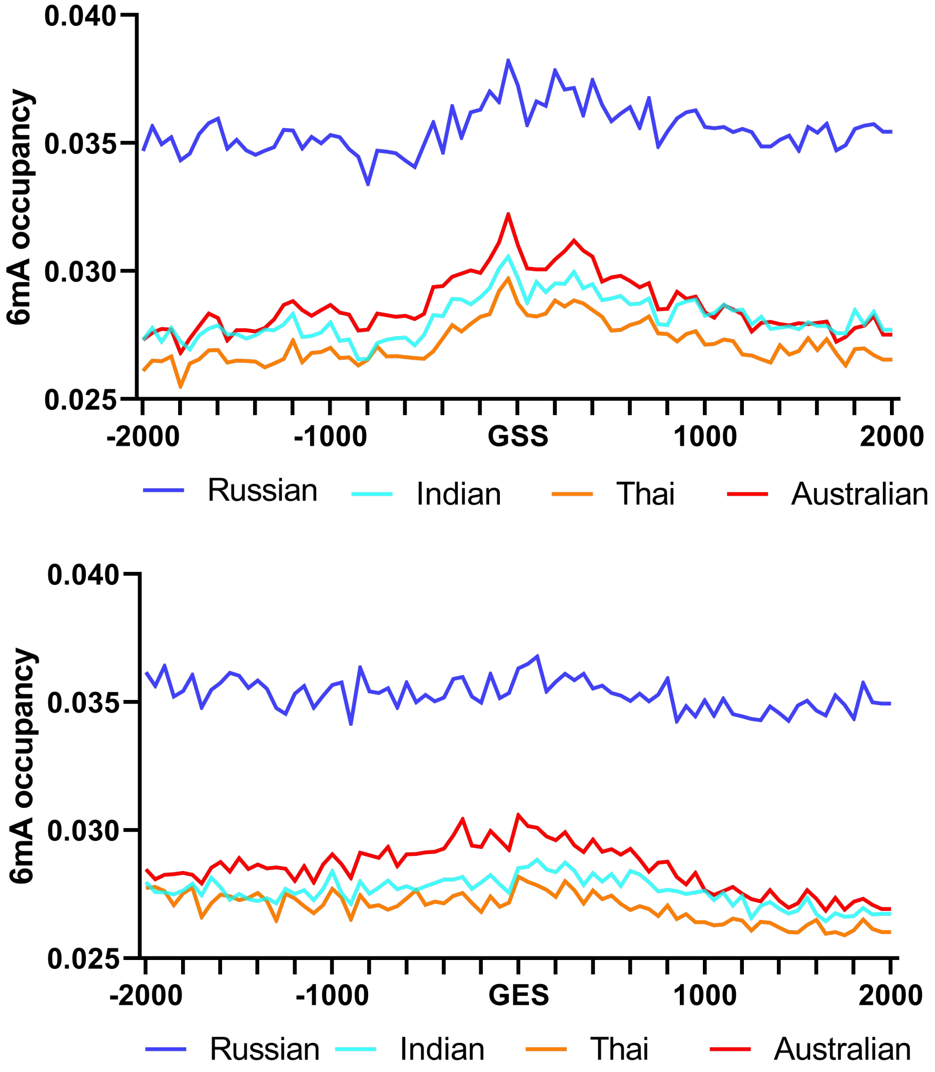

Supplementary Figure S10. Distribution of adenine bases around the GSS in four lotus genomes. Adenine occupancy represents the adenine bases out of the total bases in 50-bp sliding windows ±2 kb of the GSS.

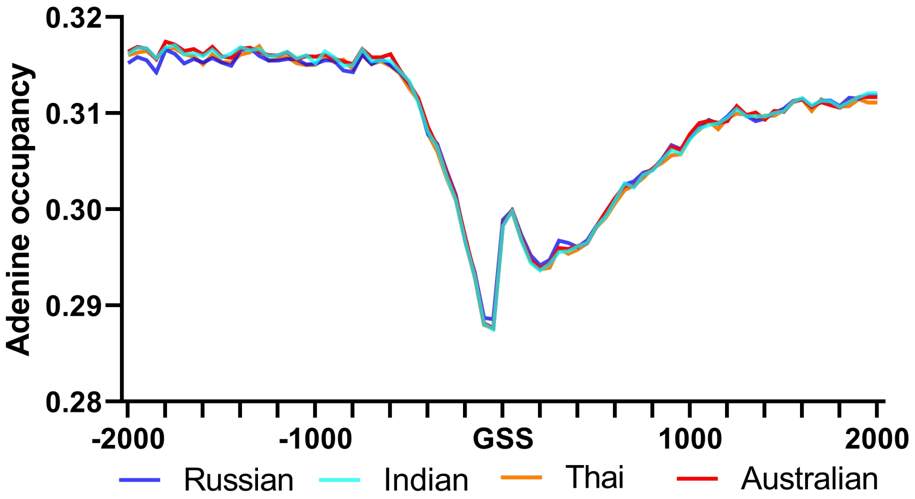

Supplementary Figure S11. Distribution of 6mA sites around transposable elements of different repeat families.

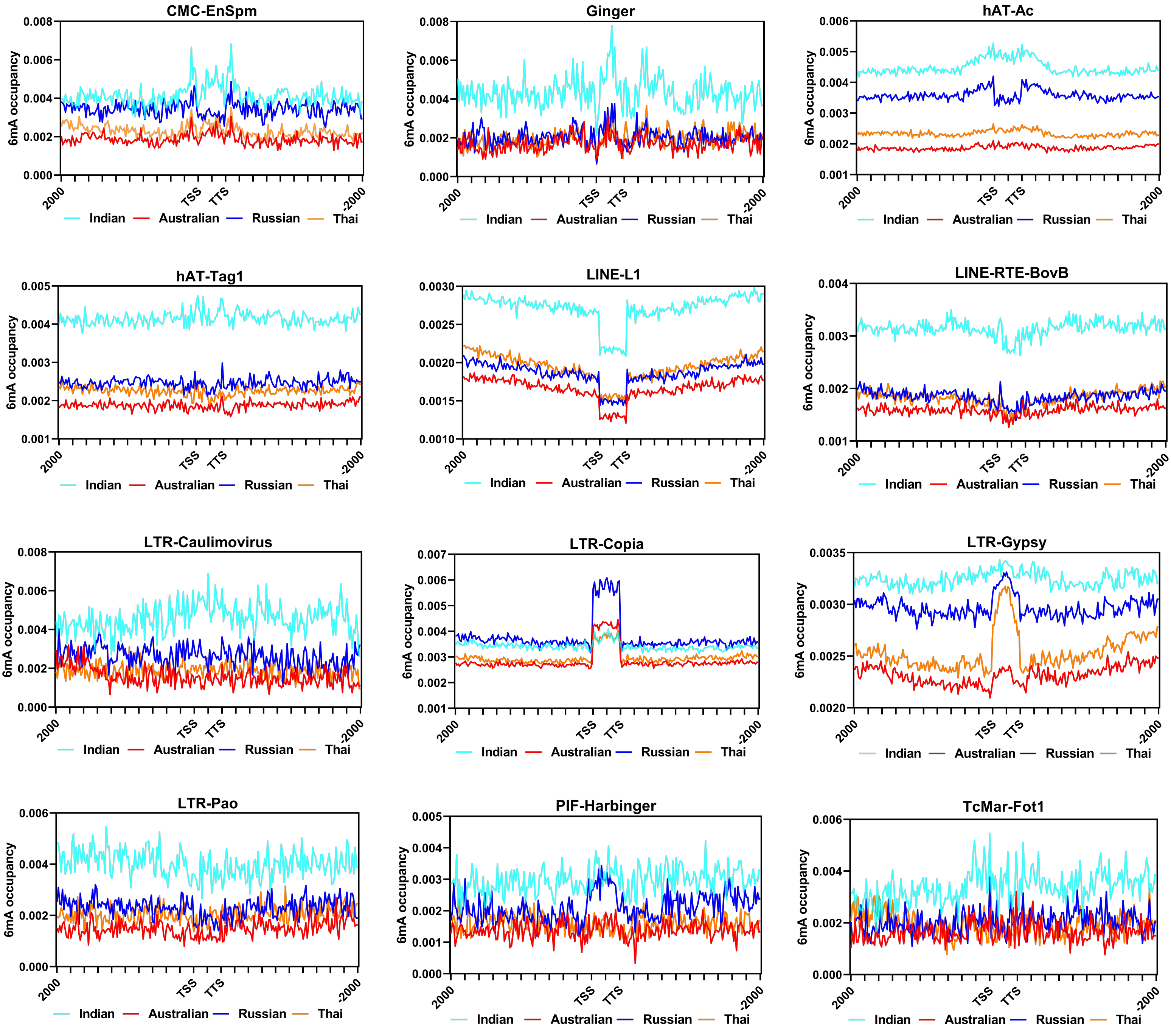

Supplementary Figure S12. Bar graph showing the most enriched functional categories in the common 6mA-methylated genes in four *N. nucifera* genomes

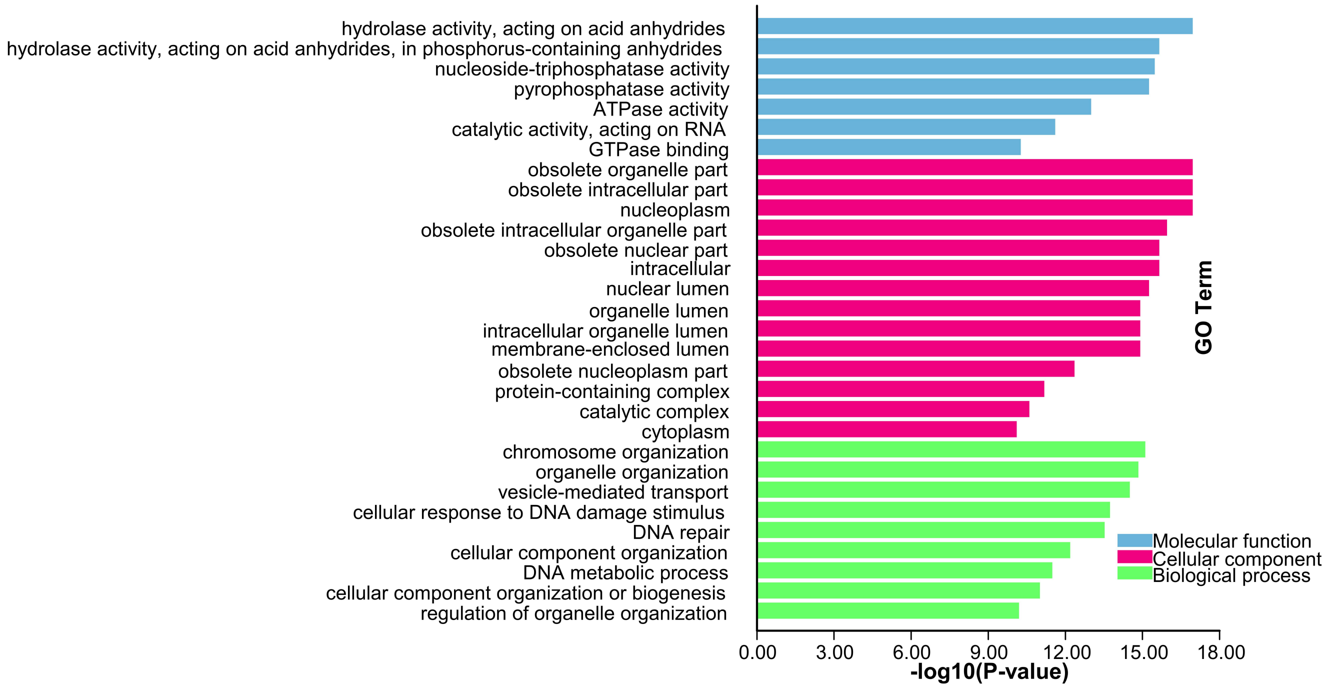

Supplement Figure S13. Distribution of gene expression level (FPKM) in genes with high 6mA methylation levels (>= 100 6mA sites), in genes with intermediate 6mA methylation levels (1~99 6mA sites), and non-6mA genes (0 6mA site). The differences of expression patterns between different gene groups were tested by Mann-Whitney U test, and ** mean p-value < 0.01.

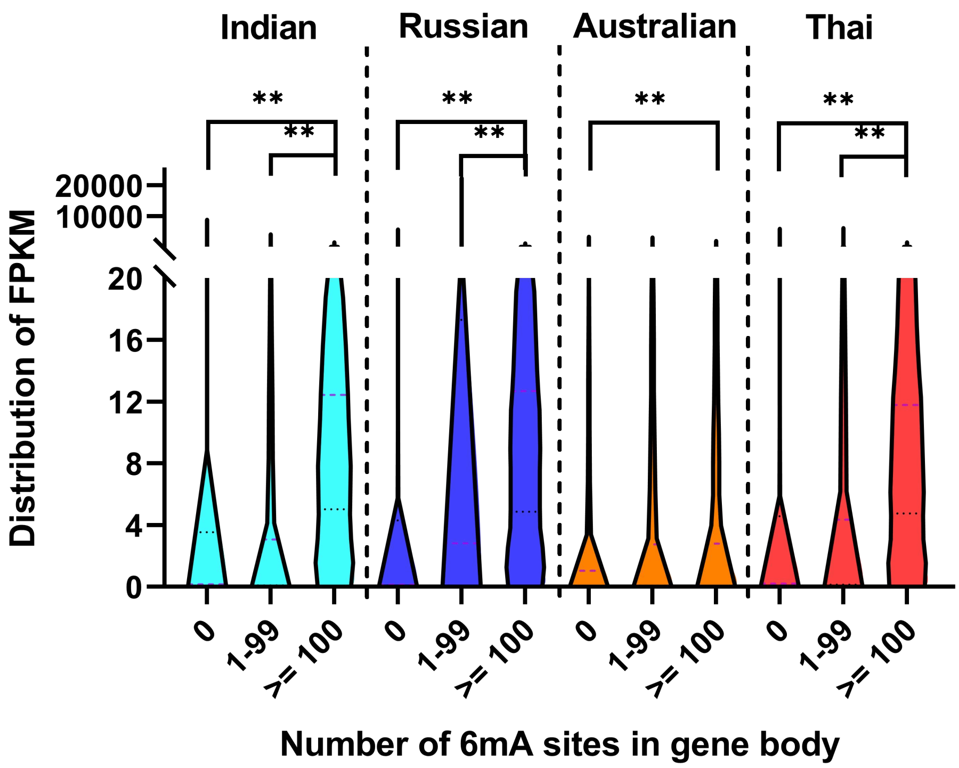

Supplementary Figure S14. Bar graphs showing the most enriched GO functional categories in the genes with high 6mA methylation levels in four lotus genomes.

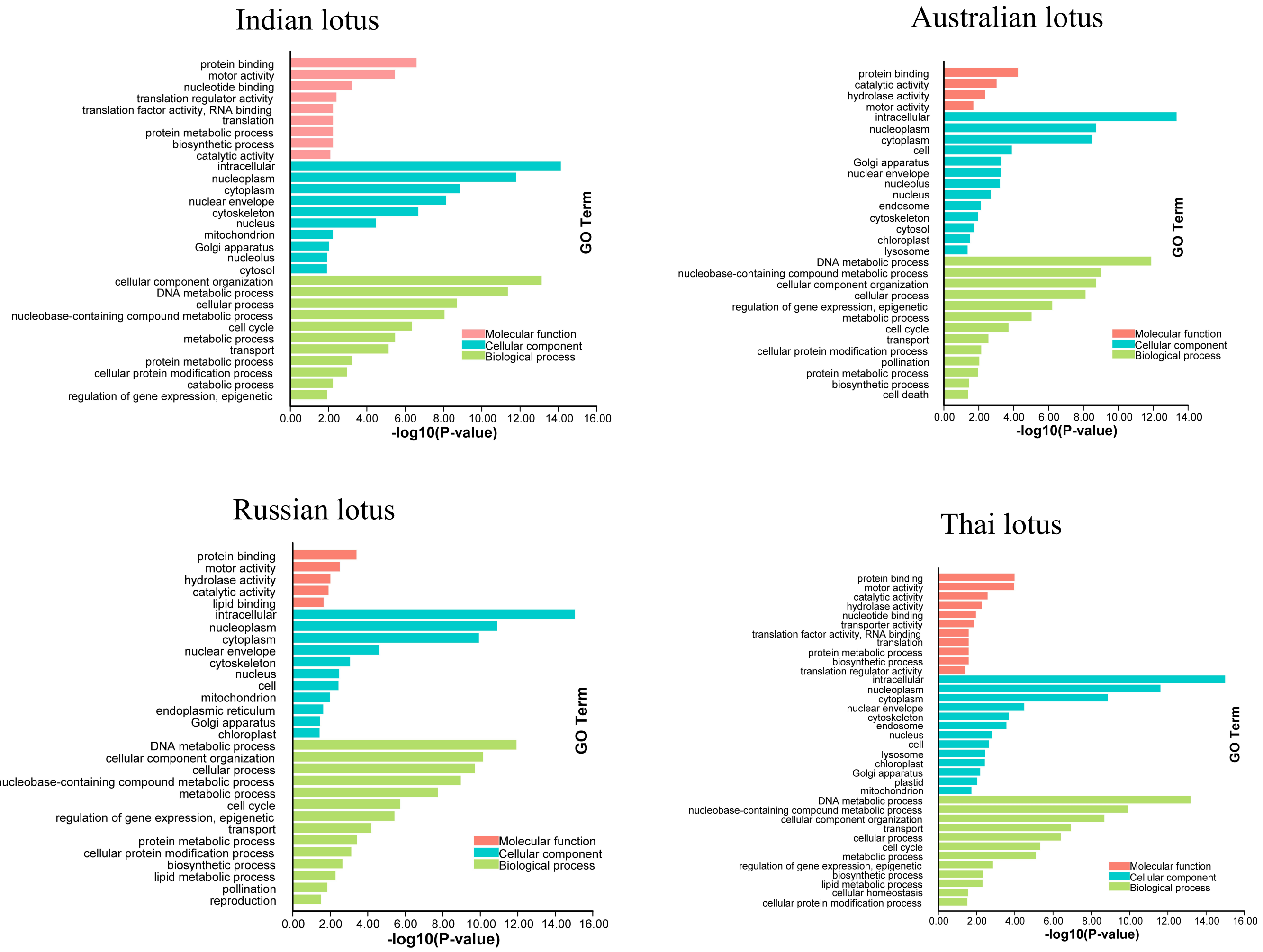

Supplement Figure S15. (A-D) Percentage of highly expressed genes (FPKM > 1, purple) and lowly expressed genes (FPKM < 1, green) in 6mA genes and non-6mA genes. The difference in the percentage of highly expressed genes between 6mA genes and non-6mA genes was examined by chi-square test. P-value < 0.01 was estimated to be significant. (E-H) Box plot comparing expression levels (FPKM) between gene promoter with and without 6mA sites in five lotus. The p-values are from two-tailed unpaired Studenst's t-test and significance was determined by p-values $< 10^{-5}$.

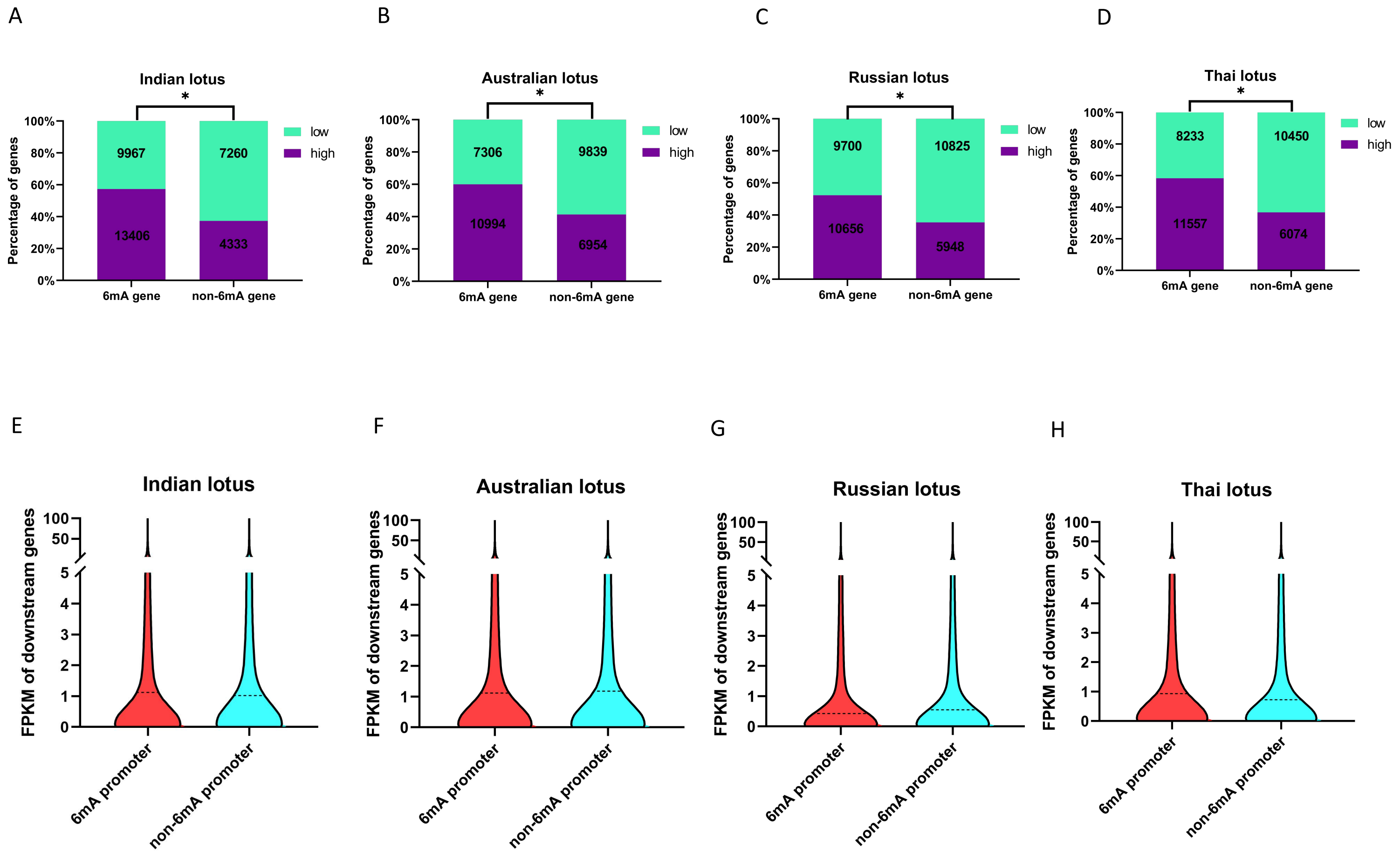

Supplementary Figure S16. Sequence features of 6mA-methylated genes in *N. nucifera*, *A. thaliana*, and *O. sativa*. The gene expression level (FPKM) (A-C) and the number of gene copies in an orthologous group (D-F) for genes with 6mA modification (w/ 6mA) or without 6mA modification (w/o 6mA). And genes of each of three species were divided into four groups from small to large referring to their quartile.

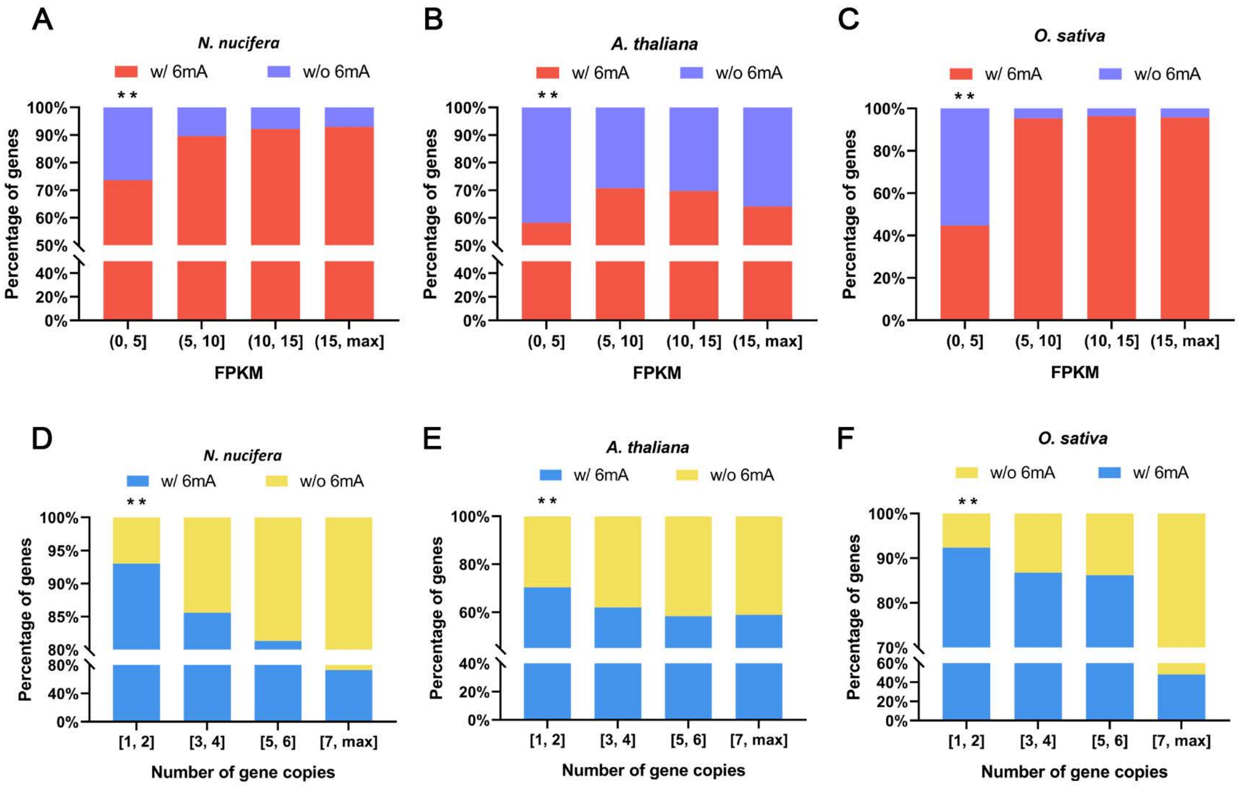

Supplementary Figure S17. Sequence features of 6mA-methylated genes on "China antique" genome chromosome1 and chromosome5. Comparative analysis of genes with 6mA modification (w/ 6mA) or without 6mA modification (w/o 6mA) in different types of sequence features, including gene length (A-B), exon number (C-D), CDS length (E-F), gene expression (G-H), and tissue-specificity (tau index) (I-J). And genes were divided into four groups from small to large referring to their quartile. The differences between different gene groups were tested by chi-square test, * was p-value < 0.05 and ** was p-value < 0.01.

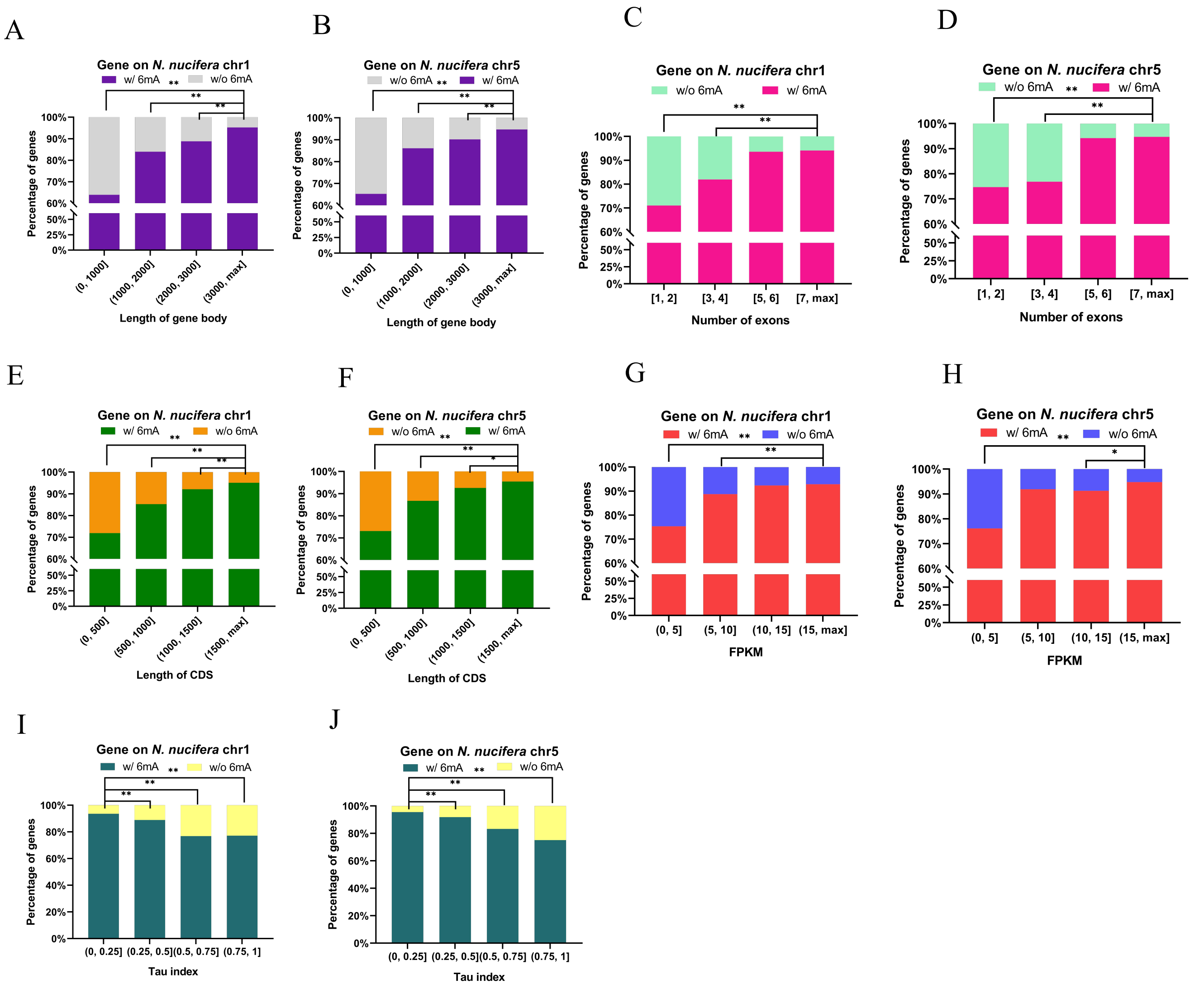

Supplementary Figure S18. Sequence features of 6mA-methylated genes in Idian lotus, Australian lotus, Russian lotus, and Thai lotus. Comparative analysis of genes with 6mA modification (w/ 6mA) or without 6mA modification (w/o 6mA) in different types of sequence features, including gene length (A-D), exon number (E-H), CDS length (I-L), gene expression (M-P), and tissue-specificity (tau index) (Q-T). And genes of each of three species were divided into four groups from small to large referring to their quartile.

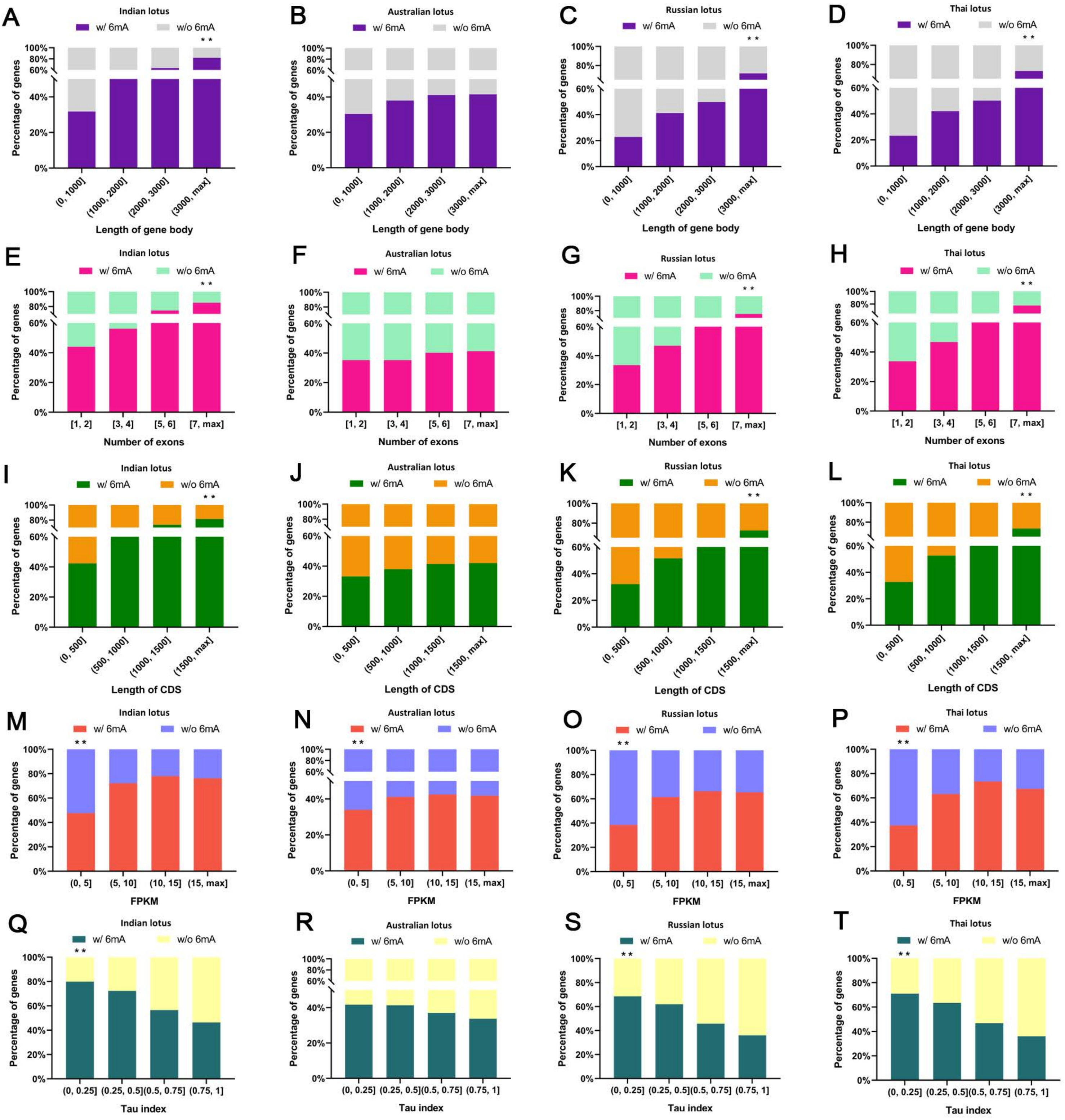

Supplementary Figure S19. Comparative analysis of genes with 6mA modification (w/ 6mA) or without 6mA modification (w/o 6mA) for the orthologous groups contained both 6mA genes and non-6mA genes in *N. nucifera*, *A. thaliana*, and *O. sativa*, respectively. The comparative analyses were carried out in different sequences features, including gene length (A), exon number (B), CDS lenght, tissue-specificity (tua index) (D), and expression level (FPKM) (E).

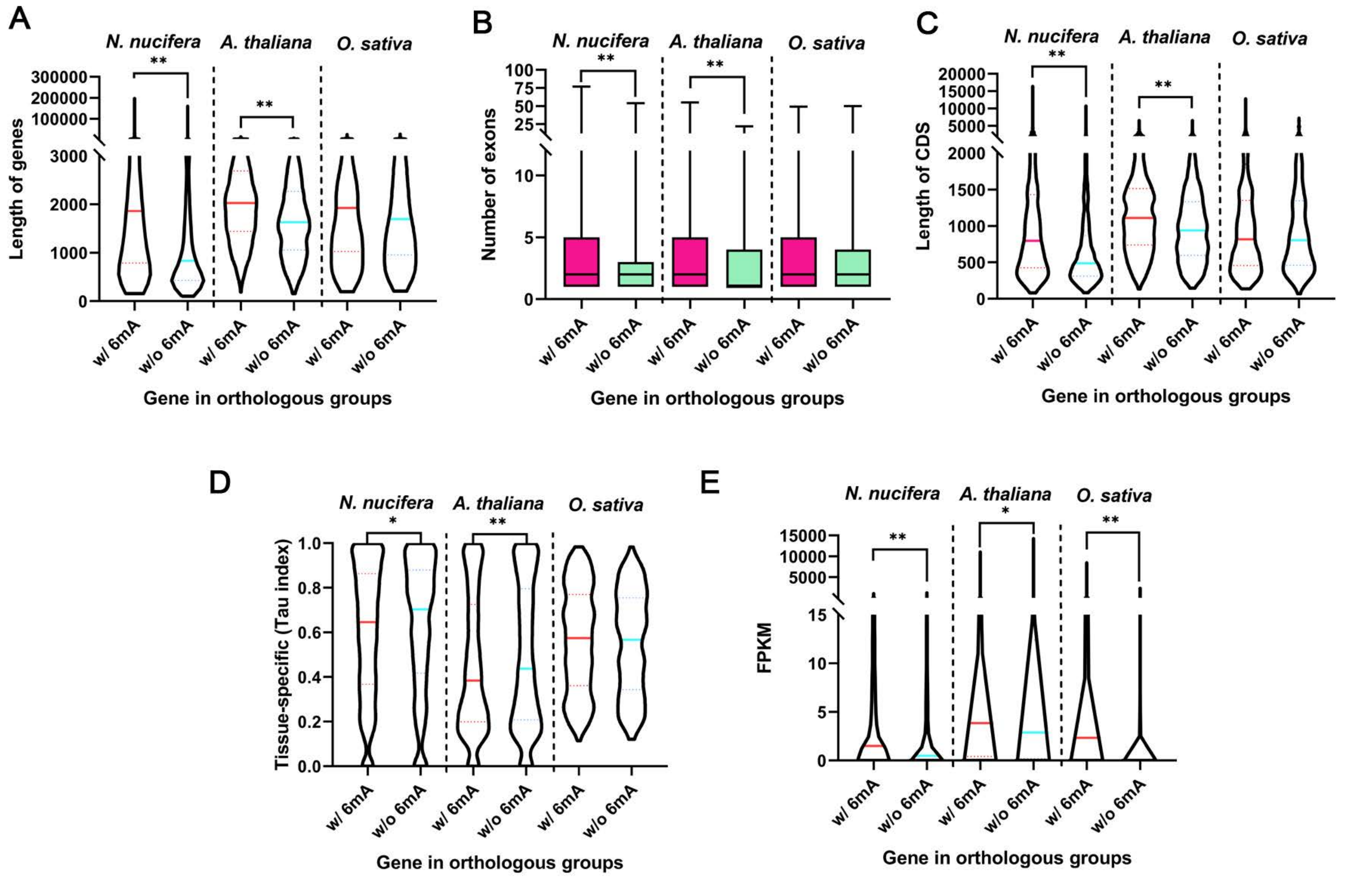

Supplementary Figure S20. Bar graph showing the most enriched GO functional categories in the orthologous genes with 6mA in *N. nucifera*, *A. thaliana*, and *O. sativa*.

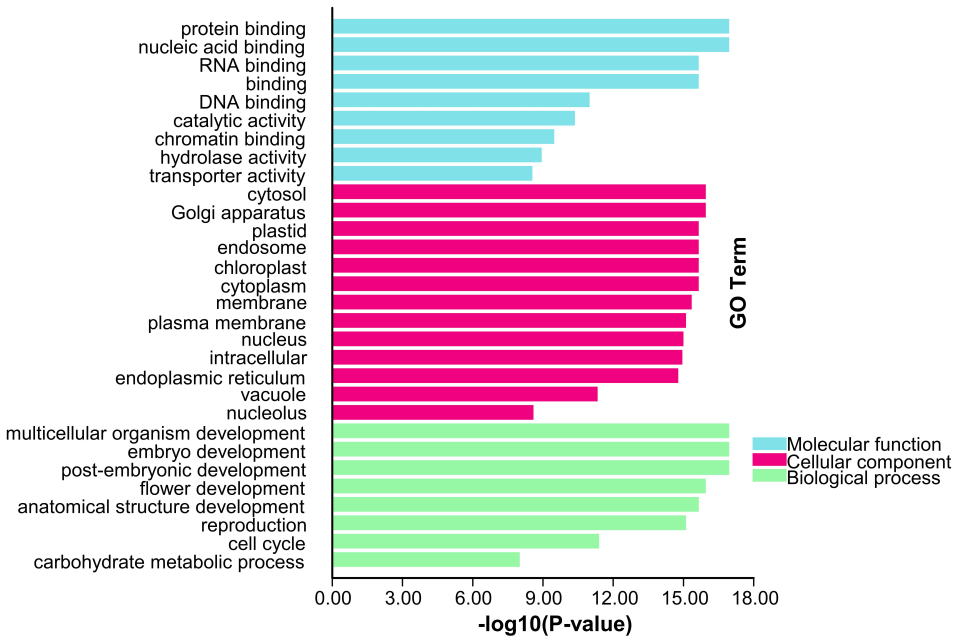

Supplement: Supplementary file 1 [file plants-12-01949-s001.zip › Supplementary Figure.pdf]
